# Supplementary material for: Influence of soil moisture levels on the growth and reproductive behaviour of Avena fatua and Avena ludoviciana
Source: PLoS One. 2020 Jul 9;15(7):e0234648. doi: 10.1371/journal.pone.0234648 (PMC7347134; doi:10.1371/journal.pone.0234648)
Supplement: Attachment — Submitted filename: Revised Response letter Plos one.docx [file pone.0234648.s003.docx]

Shahid Farooq, Ph.D.
Academic Editor
PLOS ONE

Subject: Ref.:  PONE-D-20-06038
Influence of soil moisture levels on the growth and reproductive behaviour of *Avena fatua* and *Avena ludoviciana* PLOS ONE

Dear Dr Shahid,

We thank you for the critical look on the manuscript and highly appreciate the remarks of the reviewers to improve the manuscript. The manuscript has been revised in the light of useful comments. All the comments have been incorporated in the revised version carefully. For clarity, the comments and suggestions will appear in the black colored text; while our response will appear in the blue text. In the manuscript also, specific changes can be seen in the highlighted text. We hope that the revised manuscript is now acceptable in “PLOS ONE”.

We look forward to hearing from you in due course.

Sincerely yours

Gulshan Mahajan

Editor comments

- I have received 2 reports for your submission. Both referees have positive views on the manuscript and think that the work is worth publishing. However, both have suggested several minor changes.

Thanks, the suggested changes have been incorporated.

- Both reviewers are concerned with the repeated use of Latin names of both species, although common names vary from region to region, differentiate these both species by their common names in Australia.

In Australia, both are known as black or wild oats. Therefore, we have incorpared common names used in the USA (WSSA). Also, it will be difficult to understand by readers if we use common names throughout the manuscript. Therefore, we have used Latin names throughout the text.

- More information is needed on seed collection. Whether the seeds were collected from single population? How many mother plants were there? Weed species exhibit inter and intra population differences.

The initial seeds were collected from a farmer’s field. These seeds were grown at the Gatton research farm in the next season and fresh seeds collected from those 50-60 matured plants were used for the study.

- The current version is not formatted according to Plos One.

We have now placed material and methods before results and discussion in the revised version.

- Please give due attention to all comments while revising your manuscript. I look forward to receive your revised submission.

All comments have been carefully incorporated in the revised version.

Reviewer #1: Overall, the manuscript entitled “Influence of soil moisture levels on the growth and reproductive behaviour of Avena fatua and Avena ludoviciana” presents novel and relevant results regarding the ability of these species in withstanding dryer soil conditions, a condition which is expected to become increasingly more common in the future due to ongoing global climate change.

Comment #1: It seems no information is presented regarding the actual cycle length across soil moisture levels. Is such information available?

We did not record the actual cycle length across soil moisture levels.

Comment #2: figures seem to be lacking quality, appearing blurred.

The figures have been revised again.

Line 52: scientific names of two species which are referred to as “wild oats” throughout the manuscript had already been properly introduced (line 41-42); why inform their scientific names again? This can be confusing to readers.

Because both species are different and as per journal format, we preferred scientific names rather than common names throughout the manuscript.

Line 321: this statement regarding the actual ability of crops such as wheat and barley seems vague and is not supported by any literature citation. It is clear that the authors are simply indicating this possibility, but it would be better to refer to articles (if any) which assessed these crops´ growth under water deficit. If none is found, then it seems necessary to add a sentence that “such statement remains to be evaluated” or similar.

The line has been modified as per the suggestion.

Reviewer #2: I have evaluated the manuscript "Influence of soil moisture levels on the growth and reproductive behaviour of Avena fatua and Avena ludoviciana" written by Sahil et al. Overall, it is an excellent piece of study and deserves publication.

I have a few minor suggestions (commented on the attached manuscript file).

All suggestions on the attached manuscript have been carefully incorporated.

The opening sentence of abstract defines climate change and study is on water stress. Only water stress can not be considered as climate change. Please rewrite the sentence.

The word “climate change” has been removed.

The Methods are given after results, while plos one requires methods before results.

The methods have been incorporated before the results now.


More info is needed on seed collection and storage. I suggest to add coordinates or a map showing the locations.

The required information about seed selection and storage has been added in the manuscript and coordinates have also been provided.

Authors have cited a survey work in Australia, could the current distribution map be provided in the manuscript?

This is a research paper and we think that a distribution map is not related to this study. That will be more suitable for a review paper or a book chapter.

I suggest to use common names of both species, how these are separated in Australia? The use of Latin names have been redundant throughout the manuscript. Obviously, common names will be redundantly used but better to use.

The common names of both species have been provided. In Australia, both species are known as wild/black oats; therefore, cannot differentiate. We have used common names used in the USA.
