# Supplementary material for: Influence of soil moisture levels on the growth and reproductive behaviour of Avena fatua and Avena ludoviciana
Source: PLoS One. 2020 Jul 9;15(7):e0234648. doi: 10.1371/journal.pone.0234648 (PMC7347134; doi:10.1371/journal.pone.0234648)
Supplement: Attachment — Submitted filename: Wild oat stress-graph.docx [file pone.0234648.s005.docx]

Figure 1: Maximum and minimum temperature (° C) in the screen house during the experimental study (2019)

***Avena fatua***

***a)***

***b)***

***c)***

Figure 2 a) Plant height, b) tiller number and c) leaf number per plant of *Avena fatua* at various stages of plant growth.

***Avena ludoviciana***

a)

b)

c)

Figure: 3 a) Plant height, b) tiller number and c) leaf number per plant of *Avena ludoviciana* at various stages of plant growth.
